# 2530: Should all clinical research subjects pay the same?

**DOI:** 10.1017/cts.2017.191

**Published:** 2018-05-10

**Authors:** Bernadette McKinney

**Affiliations:** University of Texas Medical Branch, Galveston, TX, USA

## Abstract

OBJECTIVES/SPECIFIC AIMS: Discuss ethical and policy issues that will impact clinical research. Raise awareness of the need to understand internal policies at home institutions Encourage further examination of ways to facilitate clinical research participation. METHODS/STUDY POPULATION: Ethical and policy analysis. RESULTS/ANTICIPATED RESULTS: Ideally, clinical research participants should not be required to pay to participate in research. However, if we go with an equity model, as opposed to an equality model, policies should be changed to allow equal access to research participation. This is a matter of justice and also will enhance the quality of the science. DISCUSSION/SIGNIFICANCE OF IMPACT: Unless steps are taken to make participation in clinical research less burdensome financially for participants, research may slow or results may be biased, because only those who can pay will be able to participate.

